# Editorial: Interdisciplinary research in diabetology

**DOI:** 10.3389/fendo.2024.1490025

**Published:** 2024-09-18

**Authors:** Nafiseh Tavasoli, Bagher Larijani

**Affiliations:** Endocrinology and Metabolism Research Center, Endocrinology and Metabolism Research Institute, Tehran University of Medical Sciences, Tehran, Iran

**Keywords:** interdisciplinary research, interdisciplinary approach, multidisciplinary medical management, metabolic disease, diabetes mellitus, diabetes care

## Introduction

Interdisciplinary research is becoming increasingly important in endocrinology, especially in the management of diabetes. The multifaceted nature of diabetes, marked by its complex etiology and various comorbidities, requires a collaborative strategy that brings together insights from multiple fields, including basic science, epidemiology, technology, and beyond. This integration not only improves our understanding of the disease and fills the gaps in our knowledge but also fosters innovative solutions that can lead to improved patient outcomes. Studies have shown that interdisciplinary collaboration can significantly improve the quality of care for patients with diabetes by addressing the intricate interplay of biological, psychological, and social factors that influence disease progression and management (1, 2). Moreover, as diabetes care evolves, the incorporation of advanced technologies, such as continuous glucose monitoring and telemedicine, highlights the importance of cross-disciplinary expertise in developing and implementing effective management strategies. The ability to work collaboratively across specialties not only enriches the research landscape but also ensures that clinical practice is informed by the latest evidence and innovations, ultimately leading to a more holistic approach to patient care. The aim of this Research Topic is to underscore the vital role of multidisciplinary research in advancing our understanding of diabetes and to promote this point of view among researchers, ultimately leading to improved health outcomes for individuals affected by this pervasive condition.

This Research Topic showcases the latest multidisciplinary research in diabetology, featuring 13 cutting-edge articles covering a wide range of topics related to type 1 and type 2 diabetes. From systematic analyses of the global burden of diabetes to innovative diagnostic methods and personalized treatment strategies, these studies underscore the vital role of collaborative, cross-disciplinary research in improving our understanding and management of this intricate disease.

## Discussion

Starting with epidemiology is crucial in a multidisciplinary approach to diabetes as it provides a foundational understanding of the disease’s prevalence, risk factors, and trends across different populations. Epidemiological studies reveal critical insights into how social determinants, lifestyle choices, and genetic predisposition contribute to the development and progression of diabetes. By identifying at-risk populations and understanding the geographic and demographic variations in diabetes incidence, researchers can tailor interventions that are culturally and contextually relevant. The global burden of diabetes is a pressing public health challenge, with the International Diabetes Federation predicting that over 1.31 billion people will be living with diabetes by 2050, driven primarily by rising obesity rates and lifestyle factors across diverse populations (3). Two articles in this Research Topic tackle the immense global burden of diabetes from different angles. Ye et al. in a systematic analysis of the Global Burden of Disease Study, provided a comprehensive overview of the past, present, and future trends in type 2 diabetes mellitus at the global, regional, and national levels. These data are critical for informing public health policy and resource allocation to combat the growing diabetes epidemic. Liang et al. focused specifically on the burden of type 1 and type 2 diabetes and high fasting plasma glucose in Europe from 1990 to 2019. By examining trends over this three-decade period, the authors shed light on the evolving nature of the disease and the need for tailored, region-specific interventions.

The incorporation of new technologies into the prevention, diagnosis, and management of diabetes marks a significant breakthrough in interdisciplinary research. Innovations such as continuous glucose monitoring systems, telehealth platforms, and artificial intelligence-driven predictive analytics are enabling more personalized and proactive care, allowing healthcare providers to tailor interventions based on real-time data and individual patient needs. These technologies not only improve clinical decision-making but also empower patients to take an active role in managing their condition, ultimately leading to better health outcomes. By fostering collaboration between technology developers, clinicians, and researchers, interdisciplinary approaches can harness the full potential of these advancements to combat the diabetes epidemic effectively. Several articles in this Research Topic showcase the potential of emerging technologies to revolutionize diabetes diagnosis and management. Litvinova et al. explored the patent landscape of digital sensors for continuous glucose monitoring, highlighting the rapid advancements in this field and the promise of improved glycemic control for patients. An et al. took a deep dive into the use of MRI fat fraction mapping to assess the association between type 2 diabetes and body composition. This non-invasive imaging technique holds promise for early detection and risk stratification, potentially leading to more targeted interventions.

Personalized medicine tailors treatment strategies based on individual patient characteristics, including genetic makeup, lifestyle, and comorbidities, requiring input from various medical disciplines such as endocrinology, genetics, nutrition, and psychology. This collaborative effort enhances the ability to identify specific biomarkers and genetic variants that predict treatment response, allowing for more effective and individualized interventions. Personalized medicine has been shown to be highly effective in diabetes management, particularly when implemented through a multidisciplinary approach. Studies show that personalized interventions that leverage the expertise of different healthcare professionals, significantly improve clinical outcomes for patients with type 2 diabetes. One study examined the practice effects of personalized interventions with interdisciplinary teamwork in achieving type 2 diabetes remission (Tian et al.). By leveraging the expertise of a diverse team of healthcare professionals, this approach demonstrates the potential for tailored, patient-centered care to yield positive outcomes. Another article delved into the relationship between illness perception and medication adherence in patients with diabetes in North Shoa, Ethiopia (Eshete et al.). Understanding the factors that influence patient behavior is crucial for developing effective, patient-centered interventions to improve treatment outcomes.

The interplay between diabetes and other conditions underscores the importance of collaboration across disciplines to better understand the origins and causes of the disease. By integrating insights from fields such as nutrition, psychology, and endocrinology, researchers can uncover the complex mechanisms that contribute to the development of diabetes. Albeloushi et al. investigated the differential effects of fish-oil and cocoa-butter-based high-fat/high-sucrose diets on endocrine pancreas morphology and function in mice, shedding light on the potential mechanisms underlying the development of diabetes. Geng et al. examined the bidirectional relationship between type 2 diabetes and major depressive disorder in a Chinese population, highlighting the importance of screening for and treating comorbid mental health conditions in diabetes patients. Wang et al. employed Mendelian randomization to investigate the causal relationship between Chronic Obstructive Pulmonary Disease (COPD) and type 2 diabetes mellitus (T2DM), revealing that COPD may serve as a significant risk factor for T2DM. Marhefkova et al. examined circadian dysfunction and its association with cardio-metabolic disorders, emphasizing the role of biological rhythms in diabetes management. Yang et al. in a narrative review focused on the methods of measuring the biomechanical properties of plantar soft tissues in patients with diabetic foot, highlighting the importance of early detection and intervention. Additionally, research by Feng et al. on the elevated triglyceride-glucose (TyG) index highlighted its association with an increased prevalence of gallstones, further illustrating the interconnectedness of metabolic disorders. Finally, Breznoscakova et al., in a case report uncovered hidden glucose patterns in patients with bipolar disorder and comorbid type 1 diabetes, underscoring the necessity of personalized approaches to treatment. Collectively, these articles underscore the multifaceted nature of diabetes and the critical need for collaborative efforts across various disciplines to improve patient care and outcomes.

## Conclusion

This Research Topic showcases the breadth and depth of multidisciplinary research in diabetology, from global epidemiology to personalized treatment approaches and the exploration of underlying mechanisms. By fostering collaboration across disciplines and borders, we can continue to advance our understanding of diabetes and develop more effective strategies for prevention and management. We hope that these articles will inspire further research and innovation in this critical field.
